# Face-gain appraisal and event-based social comparison in the association between novelty motivation and sustained positive sport experience in highland paragliding among urban professional women in China

**DOI:** 10.3389/fpsyg.2026.1877029

**Published:** 2026-06-30

**Authors:** Xiaoyun Wang, Xiangyu Wang

**Affiliations:** 1 School of Physical Education, Southwest Petroleum University, Chengdu, China; 2 Key Laboratory of Sports and Health Tourism Technology Research and Development, Department of Culture and Tourism of Sichuan Province, Chengdu, China; 3 School of Physical Education, Sichuan Normal University, Chengdu, China

**Keywords:** face-gain appraisal, highland paragliding, novelty motivation, social comparison, sport psychology, sustained positive sport experience, time-lagged indirect association, women

## Abstract

Brief adventure-sport experiences can remain psychologically meaningful after participation, but the post-event processes linking pre-event motivation with sustained positive sport experience remain under-specified. This four-wave time-lagged observational study examined whether novelty motivation before recreational highland paragliding was prospectively associated with sustained positive sport experience 1 week later, and whether this association involved two temporally ordered post-event processes: face-gain appraisal immediately after the flight and event-based social comparison during the following days. Urban professional women in China completed surveys 1–3 days before the activity, within 1 h after the flight, 24–72 h after the activity, and approximately 7 days later; the final eligible sample included 454 participants. Confirmatory factor analysis supported the distinction among the four focal constructs. Latent structural paths and baseline-covariate adjusted bootstrap models showed positive indirect associations through face-gain appraisal [(b = 0.125, 95% CI (0.083, 0.173)], event-based social comparison (b = 0.072, 95% CI [0.044, 0.105]), and the sequential appraisal-to-comparison pathway [(b = 0.031, 95% CI (0.018, 0.048)]. Sensitivity analyses adjusting for activity-context variables, attrition using inverse-probability weighting, and social-media sharing yielded the same substantive pattern. The findings indicate that sustained positive sport experience after highland paragliding is associated with pre-event novelty motivation and with the social-evaluative and comparative meanings attached to the activity after participation. Practically, the findings may inform safety-centered communication, credible recognition of accomplishment, and autonomy-supportive reflective sharing among paragliding providers, sport-tourism agencies, and women's sport organizations. The study contributes to sport psychology by conceptualizing positive adventure-sport experience as a temporally ordered motivational, appraisal-based, and comparative process. Given the observational self-report design, the results describe prospective associations rather than experimental causal effects.

## 1 Introduction

Sport and exercise psychology increasingly examines not only whether people participate in physical activity, but also how specific sport experiences become affectively rewarding, memorable, and psychologically meaningful after the activity has ended. Recent work links autonomous and intrinsic motivation with physical activity engagement, affective benefits, and subjective wellbeing (Courtney et al., 2021; Meyer et al., 2021; White et al., 2024; Meachon et al., 2025; Ntoumanis and Moller, 2025). These motivational processes unfold within social contexts and temporal sequences rather than as isolated individual traits. This perspective is especially relevant for outdoor and adventure sport, where participants often seek novelty, challenge, embodied confidence, emotional intensity, and social meaning alongside technical performance.

Highland paragliding offers a focused context for examining these processes. In this study, highland paragliding refers to recreational tandem paragliding conducted at mountain or highland tourism sites rather than to laboratory-based motor training or physiological altitude exposure. The activity is brief, physically distinctive, memorable, socially visible, and risk-managed. All activities in the study were recreational tandem flights conducted at five commercial mountain or highland tourism sites under instructor-guided conditions; solo training flights, competitive paragliding, and laboratory-based altitude exposure were not included. Adventure and active sport tourism experiences have been associated with subjective wellbeing, psychological recovery, recollection, storytelling, and post-trip engagement (Hung and Wu, 2021; Su et al., 2021, 2022, 2023; So et al., 2024; Yan et al., 2024; Song et al., 2025). From a sport psychology perspective, however, the processes through which pre-event novelty motivation is linked with sustained positive sport experience after participation remain insufficiently specified.

The present study addresses this gap in a theoretically specific population: urban professional women in China. In this manuscript, urban professional women are adult women who self-identified as occupationally engaged and met the study's urban-sample eligibility criterion. This population is relevant for three reasons. First, women remain unevenly represented in parts of sport and exercise psychology evidence, making female-focused designs valuable when they are theoretically justified (Walton et al., 2024). Second, studies of women and physical activity show that intention, social comparison, and daily appraisal can shape affective and behavioral responses to exercise (Arigo et al., 2022; Baga et al., 2024; Arigo et al., 2025). Third, women-centered tourism and leisure research suggests that travel experiences can carry meanings related to autonomy, identity, care, companionship, and wellbeing (Kong et al., 2022; Vespestad, 2023; Wang et al., 2023). Together, this evidence identifies urban professional women as an informative population for examining social-evaluative appraisal and post-event meaning in highland paragliding.

Novelty motivation refers to the desire to encounter something new, unfamiliar, distinctive, or different from ordinary routines. In tourism and leisure research, novelty is a central motivational and appraisal dimension: new experiences can activate interest, emotion, memorability, and post-trip engagement (Chen et al., 2021; He et al., 2021; Blomstervik and Olsen, 2022; Zhang and Deng, 2022; Yan et al., 2024; Blomstervik and Olsen, 2026). In sport psychology terms, novelty can increase the subjective value of participation by making the activity feel personally chosen, emotionally vivid, and meaningfully different from everyday exercise or recreation. Recent evidence also suggests that exercise motivation and physical activity can relate to subjective wellbeing through sequential psychological pathways rather than through a single direct route (Feng et al., 2024; Liu, 2025; Liu et al., 2025; Zhang et al., 2025).

For highland paragliding, novelty motivation is theoretically relevant because the activity combines unusual movement, altitude, scenery, perceived challenge, and a strong first-person narrative. Novelty alone is not always sufficient for a positive experience to endure. A participant can feel excited by a novel flight but later reinterpret the experience as frightening, ordinary, or socially irrelevant. A complete sport psychology model therefore needs to explain how novelty is appraised and socially processed after the activity. This study proposes face-gain appraisal and event-based social comparison as two temporally ordered post-event processes.

Face-gain appraisal refers to interpreting the paragliding experience as enhancing social recognition, self-presentation, confidence in front of others, or symbolic accomplishment. In Chinese social contexts, face-related appraisals are tied to social self-image, recognition, and culturally embedded evaluation. Recent research on face and consumption in China shows that face-related motives and regulatory foci can shape attitudes and intentions in domains where behavior is socially meaningful and normatively visible (Shi and Chen, 2024; Wei and Shen, 2025). In tourism and leisure settings, experiences are frequently shared, narrated, and evaluated through social media and interpersonal storytelling, making social visibility part of the event's post-activity psychological meaning (Chen et al., 2021; Su et al., 2021; Wang et al., 2023). Although the term face-gain is culturally situated, the psychological function it captures overlaps with broader social-evaluative processes such as self-presentation, identity signaling, reputational recognition, and impression management.

Highland paragliding is well suited to face-gain appraisal because it can be interpreted as involving bravery, distinctiveness, aesthetic impressiveness, and personal accomplishment. Beyond completing the activity, participants can acquire a socially communicable account of achievement. When a novel experience is immediately appraised as face-enhancing, this appraisal can help preserve positive emotional meaning across the following week. Face-gain appraisal therefore offers one pathway linking novelty motivation with sustained positive sport experience.

Social comparison is another plausible process linking novelty motivation with sustained experience. After visible sport and travel activities, participants can compare their own experience with those of friends, colleagues, online peers, or other participants. Social comparison can amplify identification, pride, inspiration, or contrast depending on perceived similarity and comparison direction (Perey and Koenigstorfer, 2023; Xu et al., 2023; Baga et al., 2024; Arigo et al., 2025). In women-focused physical activity research, comparison processes are not merely ancillary; they can shape affective interpretation and motivational meaning after exercise or sport participation (Baga et al., 2024; Arigo et al., 2025).

In the present study, event-based social comparison is conceptualized as a post-activity evaluation process in which participants compare the meaning, distinctiveness, bravery, or social value of their paragliding experience with relevant others. Such comparison provides a frame through which a single sport event remains personally and socially interpretable. Social comparison can also follow from face-gain appraisal: if a participant sees the flight as face-enhancing shortly after the activity, she can later compare the experience with what others have done, how others would evaluate it, or how it positions her in a social reference group. Thus, face-gain appraisal is positioned as an immediate social-evaluative appraisal, whereas event-based social comparison is positioned as a later comparison-based meaning-making process.

**Figure 1:**
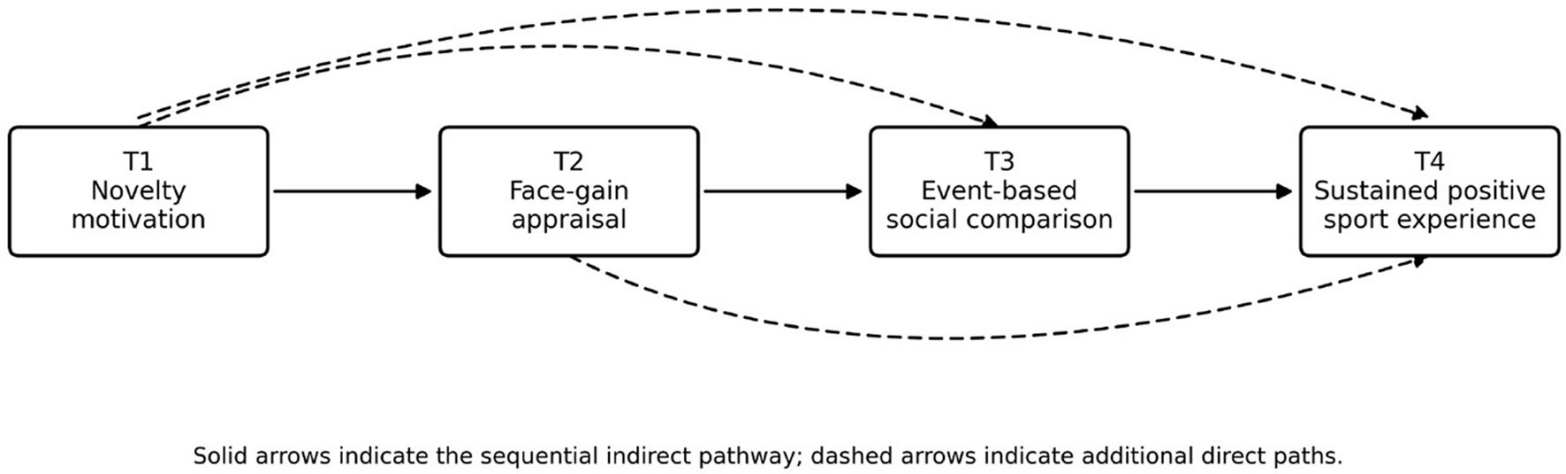
Conceptual four-wave sequential indirect-association model. Solid arrows indicate the sequential path from T1 novelty motivation to T2 face-gain appraisal, T3 event-based social comparison, and T4 sustained positive sport experience. Dashed arrows indicate additional direct paths. H1 refers to the direct association between novelty motivation and sustained positive sport experience; H2-H4 refer to the indirect paths stated in the Introduction.

The present study tested a four-wave sequential indirect-association model in which T1 novelty motivation was hypothesized to be associated with T4 sustained positive sport experience through T2 face-gain appraisal and T3 event-based social comparison (Figure 1). Temporal separation reduced same-time method concerns and aligned measurement timing with the proposed psychological sequence. The design supports prospective ordering while remaining observational; causal claims require experimental or quasi-experimental evidence. Recent methodological literature similarly emphasizes that common method variance is not resolved by any single procedural choice, so longitudinal and time-separated self-report designs benefit from sensitivity checks (Baumgartner et al., 2021; Baumgartner and Weijters, 2021; Kaltsonoudi et al., 2022; Sturman et al., 2025).

The hypotheses were:

H1: T1 novelty motivation will be positively associated with T4 sustained positive sport experience.

H2: T1 novelty motivation will show a positive indirect association with T4 sustained positive sport experience through T2 face-gain appraisal.

H3: T1 novelty motivation will show a positive indirect association with T4 sustained positive sport experience through T3 event-based social comparison.

H4: T1 novelty motivation will show a positive sequential indirect association with T4 sustained positive sport experience through T2 face-gain appraisal and T3 event-based social comparison.

## 2 Materials and methods

### 2.1 Design and setting

This study used a four-wave time-lagged observational survey design. Data were collected from women participating in highland paragliding activities in China between October 1 and December 10, 2025. The four waves were aligned with the activity timeline: T1 was completed 1–3 days before the activity, T2 within 1 h after the flight, T3 24–72 h after the activity, and T4 approximately 7 days later. This structure separated the focal predictor, first intermediate process, second intermediate process, and outcome in time and supports prospective ordering of constructs; causal effects require experimental manipulation.

### 2.2 Participants and recruitment

Participants were urban professional women who had registered for a highland paragliding experience and completed the activity during the study period. Eligibility required participants to identify as female, be at least 18 years old, self-report current occupational engagement, meet the study's urban-sample criterion, participate in a highland paragliding activity during the data-collection window, and agree to be contacted for follow-up surveys. Operationally, the urban professional criterion combined self-reported residence or work in an urban city category with current occupational engagement in enterprise management or professional-technical work, finance, internet, media, education, medical or public service, self-employment or entrepreneurship, or another paid occupation. Respondents without current occupational engagement were not eligible. Participants were recruited through cooperating highland paragliding sites and activity-administration channels during the survey window. Anonymous matching across waves used a self-generated participant code rather than directly identifying information; names, identity numbers, phone numbers, and precise residential addresses were not retained in the analytic dataset. Participants who did not pass all required attention checks across the four waves were excluded from the main analysis sample.

The merged dataset initially included 700 T1 respondents. Of these, 657 completed T2, 588 completed T3, and 475 completed T4. After excluding 21 participants who did not pass all four required attention checks among the four-wave completers, the final eligible sample included 454 women. Their mean age was 31.36 years (SD = 6.13, range = 18–48). Most participants reported that the activity was their first paragliding experience (n = 317, 69.8%). The mean flight duration was 16.91 min (SD = 6.63), and the mean T4 follow-up interval was 8.00 days (SD = 0.83). Counts represent observed completed records at each wave and were not rounded.

### 2.3 Analytic sample definitions

Sample size varied across analyses because different analyses required different levels of data completeness. The final eligible sample was *N* = 454 after attention-check exclusions. Scale reliability analyses used scale-specific complete item data, resulting in *n* = 426-445 across constructs. The CFA, HTMT, and latent structural analyses required complete item-level data across all 26 focal items, resulting in *n* = 385. The adjusted composite-score indirect-association models used complete focal composite and covariate data, resulting in *n* = 454. These analytic samples are reported separately to distinguish item-level completeness from composite-level completeness.2.4

### Ethics statement

This study followed the ethical principles of the Declaration of Helsinki. Under Article 32(2) of the Measures for Ethical Review of Life Sciences and Medical Research Involving Humans issued by the National Health Commission of the People's Republic of China, the Ministry of Education, the Ministry of Science and Technology, and the National Administration of Traditional Chinese Medicine, research using anonymized information data may be exempt from formal ethical review when it does not cause harm to human participants and does not involve sensitive personal information or commercial interests (National Health Commission of the People's Republic of China et al., 2023). Before data analysis, the authors documented the exemption basis according to this regulation. The study met the exemption conditions: it used an anonymous voluntary questionnaire survey of adult urban professional women participating in highland paragliding, involved no clinical intervention, biological samples, human experimentation, minors, identifiable personal information, sensitive personal information, or commercial interests, and all data were de-identified before analysis. At the beginning of each survey wave, participants were informed of the study purpose, anonymous matching procedure, voluntary nature of participation, right to withdraw, and intended use of the data. Electronic informed consent was obtained from all participants before questionnaire completion.

### 2.4 Measures

All focal variables were measured with multi-item Likert-type scales adapted to the highland paragliding context and administered in Chinese. Items used a 7-point response format ranging from 1 = strongly disagree to 7 = strongly agree; higher scores indicated higher levels of the target construct. Composite scores were calculated as scale means. Items were reviewed for contextual relevance, temporal fit with the four-wave design, and conceptual alignment with the sport psychology model. English translations were retained only for reporting. The instruments were treated as contextualized measures for this study. Full item wording, response anchors, adaptation procedures, scoring rules, and content-validation information are reported in Supplementary Appendix A.

T1 novelty motivation was measured with six items assessing the extent to which participants were motivated by newness, distinctiveness, unfamiliarity, and the wish to experience something different through highland paragliding. This construct was grounded in recent novelty and tourism-motivation literature (Blomstervik and Olsen, 2022, 2026).

T2 face-gain appraisal was measured with six items assessing whether the activity was appraised as enhancing social image, recognition, confidence in front of others, and socially valued self-presentation. The measure was developed for this sport-tourism context based on recent work on face-related appraisal and social image in Chinese settings (Shi and Chen, 2024; Wei and Shen, 2025).

T3 event-based social comparison was measured with six items assessing post-activity comparison with relevant others regarding bravery, distinctiveness, experience quality, and social meaning. The construct was based on recent physical activity and tourism social-comparison research (Perey and Koenigstorfer, 2023; Xu et al., 2023; Baga et al., 2024; Arigo et al., 2025).

T4 sustained positive sport experience was measured with eight items assessing the extent to which the paragliding experience remained positive, memorable, meaningful, enjoyable, and psychologically energizing approximately 1 week after the activity. This outcome aligns with recent evidence that sport, active tourism, and travel experiences can relate to subjective wellbeing through enjoyment, recollection, engagement, and psychological recovery (Su et al., 2023; So et al., 2024; White et al., 2024; Song et al., 2025; Meachon et al., 2025).

The primary baseline-covariate adjusted models included age, education, income, career level, prior paragliding experience, prior adventure sport experience, sport participation frequency, sensation seeking, risk perception, baseline positive affect, baseline face concern, and baseline social comparison orientation. Demographic and experience covariates were self-reported at T1. Sensation seeking, risk perception, baseline positive affect, baseline face concern, and baseline social comparison orientation were administered as brief baseline covariate indices; item wording, response anchors, and scoring rules are reported in Supplementary Appendix A. Expanded sensitivity models additionally included activity-context variables: immediate enjoyment, perceived safety, flight duration, weather satisfaction, and coach service. A supporting social-visibility sensitivity model additionally adjusted the T3 social-comparison equation and the T4 outcome equation for whether participants reported sharing the experience on social media. Because social-media sharing and several activity-context variables occurred after the focal T1 exposure and can overlap with post-event appraisal or meaning-making processes, these variables were used in sensitivity models rather than primary confounder controls.

### 2.5 Statistical analysis

Analyses were conducted with Python using pandas, NumPy, SciPy, scikit-learn, and statsmodels. The analysis code, cleaned analytic dataset, diagnostics, and table-generation files are described in Supplementary Appendix B. First, sample flow, descriptive statistics, and attrition patterns were examined. Internal consistency was assessed using Cronbach's alpha and McDonald's omega estimated from a one-factor model. Composite reliability and average variance extracted were calculated for the four focal constructs. Discriminant validity was evaluated using heterotrait-monotrait ratios. Reliability coefficients were computed from scale-specific complete item data, whereas the CFA and HTMT analyses used participants with complete item-level data across all four focal scales.

Second, a four-factor confirmatory factor analysis (CFA) was estimated for novelty motivation, face-gain appraisal, event-based social comparison, and sustained positive sport experience. The 7-point Likert-type items were treated as approximately continuous, a decision supported by the response scale length and the study's focus on latent structure rather than individual ordinal thresholds. The CFA was estimated using a maximum-likelihood covariance-fitting routine implemented in Python; convergence information, package versions, and reproducibility files are listed in Supplementary Appendix B. Model fit was evaluated using chi-square, CFI, TLI, RMSEA with 90% confidence interval, SRMR, and related indices. Because fit-index thresholds can be model- and context-sensitive, fit was interpreted using multiple indicators rather than a single mechanical cutoff (McNeish and Wolf, 2023; Goretzko et al., 2024).

Given the close fit of the final four-factor model, additional plausibility checks compared the hypothesized correlated four-factor solution with one-factor, two-factor, and orthogonal four-factor alternatives. Pairwise item correlations, exact-response equality rates between item pairs, residual correlations, and random split-half fit were also inspected to evaluate whether the result plausibly reflected a clean simple structure rather than duplicated items or a single general factor.

Third, standardized latent structural path estimates were derived from the fitted four-factor latent covariance matrix to test the proposed paths among the four focal constructs. The latent analysis used complete item-level data across all 26 focal items (*n* = 385). The structural portion of the hypothesized latent model was saturated among the four latent constructs needed for the sequential indirect-association sequence. Therefore, global model fit was expected to match the four-factor measurement model, and substantive interpretation focused on path estimates rather than on global fit as evidence of structural-model superiority. Fourth, primary adjusted composite-score indirect-association models were estimated with baseline covariates, heteroskedasticity-consistent standard errors, and 5,000 bootstrap resamples for indirect associations. These models used complete focal composite and covariate data (*n* = 454). Focal path coefficients, HC3 standard errors, 95% confidence intervals, *p*-values, and equation R-squared values were reported before the bootstrap indirect associations.

Additional diagnostics were conducted to evaluate statistical reliability. Missingness in the adjusted composite model variables was inspected before complete-case estimation, and the main focal analyses did not use model-based imputation. Multicollinearity was assessed with variance inflation factors. Potentially influential observations were examined using Cook's D, and multivariate outliers in the four focal composites were examined using Mahalanobis distance with a chi-square.999 cutoff. Sensitivity models then re-estimated the adjusted composite path estimates after excluding influential observations, multivariate outliers, or both. Correlation analyses and diagnostic checks were treated as descriptive or supporting analyses; the focal hypothesis tests were the theoretically specified path and indirect-association tests. A sample-size sensitivity calculation was also used to estimate the smallest detectable regression associations under the observed analytic sample size. Exact software and package versions are listed in Supplementary Appendix B.

Finally, inverse-probability weighting (IPW) was used to assess attrition sensitivity because attrition between T1 and T4 was associated with observed baseline characteristics. Completion propensity was estimated from T1 demographic, experience, and baseline psychological variables, and trimmed stabilized weights were applied to the adjusted path models. Weighted bootstrap confidence intervals were estimated for the key indirect and direct associations. The latent SEM was used to test the measurement-informed structural pattern, whereas the adjusted composite-score models were used for covariate-adjusted indirect-association inference. Longitudinal indirect-pathway analysis in non-randomized designs is sensitive to unmeasured confounding and model misspecification; the estimates are therefore reported as prospective indirect associations rather than definitive causal mechanisms (Tofighi, 2021; Tai et al., 2022).

Social visibility was theoretically relevant, so an additional supporting sensitivity analysis adjusted the T3 social-comparison equation and the T4 sustained positive sport experience equation for whether participants reported sharing the experience on social media. This analysis was not treated as a primary confounder adjustment because sharing occurred after the focal T1 exposure and can itself be part of the post-event meaning-making process.

## 3 Results

### 3.1 Sample characteristics and attrition

Table 1 summarizes the main analysis sample. The analytic sample included 454 women, with a mean age of 31.36 years. Participants came from first-tier or new first-tier cities, provincial capital cities, prefecture-level cities, and other cities. Five paragliding sites were represented. Approximately two-thirds of participants were first-time paragliding participants, and slightly more than half reported sharing the experience on social media.

**Table 1:** Participant characteristics in the main analysis sample (*N* = 454).

Characteristic	*N* (%) or *M* (SD)
Age, years	31.36 (6.13)
Flight duration, minutes	16.91 (6.63)
Follow-up interval, days	8.00 (0.83)
First paragliding experience: yes	317 (69.8%)
First paragliding experience: no	137 (30.2%)
Shared the experience on social media: yes	239 (52.6%)
Shared the experience on social media: no	215 (47.4%)
Married: yes	198 (43.6%)
Married: no	256 (56.4%)
Has child: yes	102 (22.5%)
Has child: no	352 (77.5%)
First-tier/new first-tier city	168 (37.0%)
Provincial capital city	137 (30.2%)
Prefecture-level city	109 (24.0%)
Other city	40 (8.8%)
Paragliding site S01	106 (23.3%)
Paragliding site S02	83 (18.3%)
Paragliding site S03	127 (28.0%)
Paragliding site S04	78 (17.2%)
Paragliding site S05	60 (13.2%)

Attrition was associated with observed baseline characteristics. Compared with participants who did not complete T4, T4 completers had higher T1 novelty motivation (*M* = 5.39 vs. 4.22, *p* < 0.001, standardized difference = 0.95) and higher sensation seeking (*M* = 4.85 vs. 4.12, *p* < 0.001, standardized difference = 0.56). They also had higher income (*p* < 0.001, standardized difference = 0.46), baseline positive affect (*p* < 0.001, standardized difference = 0.46), education (*p* < 0.001, standardized difference = 0.39), baseline face concern (*p* < 0.001, standardized difference = 0.37), and career level (*p* < 0.001, standardized difference = 0.35). Attrition sensitivity results therefore follow.

### 3.2 Reliability, validity, and measurement model

**Figure 2:**
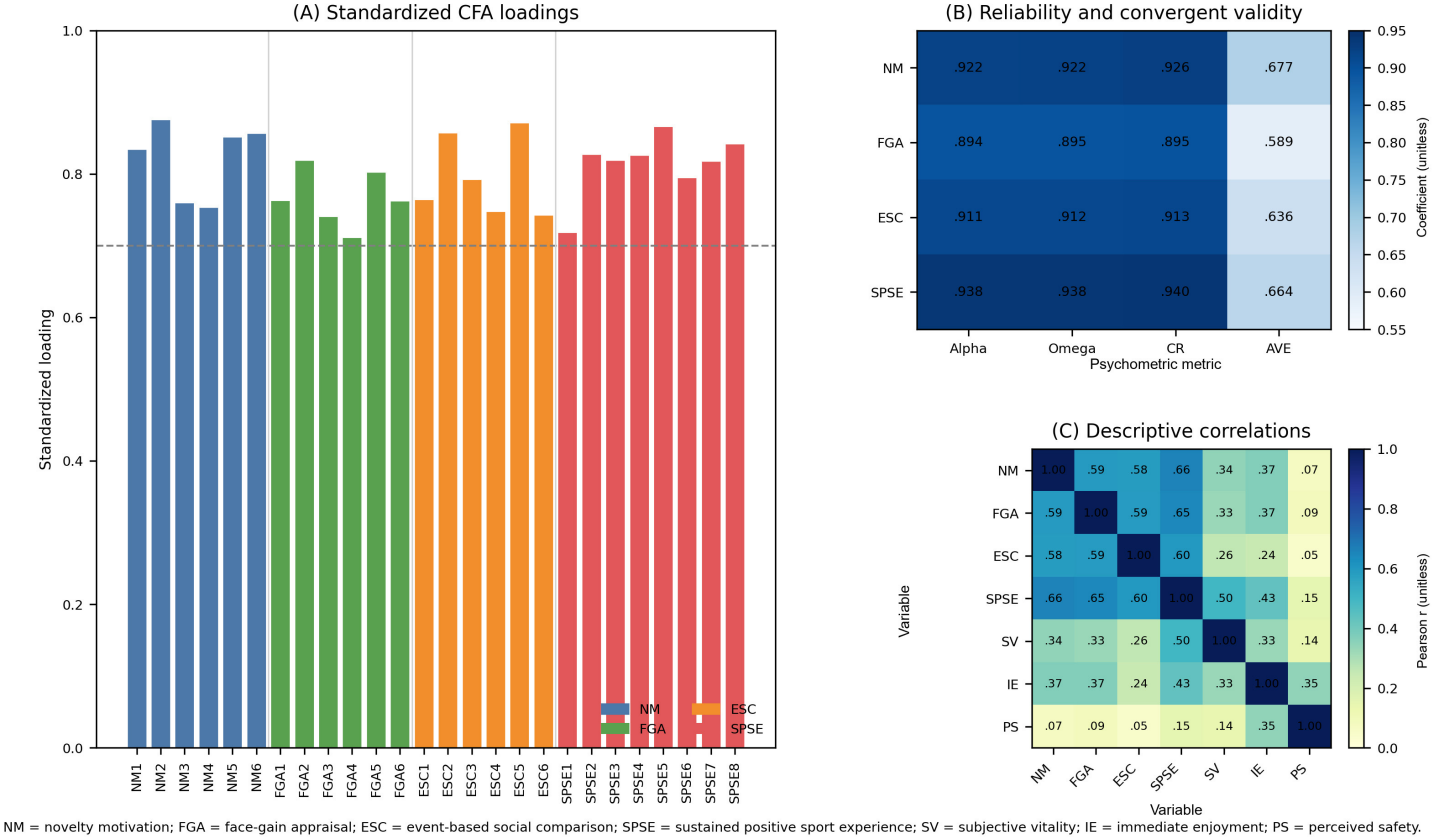
Measurement quality and descriptive associations. **(A)** Standardized CFA loadings for the 26 focal items; the dashed vertical reference line marks.70. **(B)** Internal consistency and convergent-validity coefficients for novelty motivation, face-gain appraisal, event-based social comparison, and sustained positive sport experience. **(C)** Descriptive correlations among focal and validation variables. NM, novelty motivation; FGA, face-gain appraisal; ESC, event-based social comparison; SPSE, sustained positive sport experience; SV, subjective vitality; IE, immediate enjoyment; PS, perceived safety.

All focal scales showed strong internal consistency and convergent validity (Table 2). Cronbach's alpha ranged from.894 to.938, omega ranged from.895 to.938, composite reliability ranged from.895 to.940, and AVE ranged from.589 to.677. Scale-specific complete item *n* ranged from 426 to 445; the CFA and HTMT sample requiring complete data across all 26 focal items was *n* = 385. All HTMT values were below 85. Figure 2 summarizes the standardized item loadings, reliability and convergent-validity coefficients, and descriptive correlations among the focal and validation variables.

**Table 2:** Reliability and validity evidence for focal constructs.

Construct	Items	Complete item n	Alpha	Omega	CR	AVE
Novelty motivation	6	445	0.922	0.922	0.926	0.677
Face-gain appraisal	6	433	0.894	0.895	0.895	0.589
Event-based social comparison	6	432	0.911	0.912	0.913	0.636
Sustained positive sport experience	8	426	0.938	0.938	0.940	0.664

The correlated four-factor CFA showed close global fit and supported the empirical distinction among novelty motivation, face-gain appraisal, event-based social comparison, and sustained positive sport experience. Standardized loadings ranged from 712 to 876. Comparative fit indices favored the hypothesized four-factor model over one-factor, two-factor pre/post, and orthogonal four-factor alternatives (Table 3).

**Table 3:** Comparative fit indices for alternative measurement models.

Model	*χ^2^*	df	*p*	CFI	TLI	RMSEA [90% CI]	SRMR	Fit
Correlated four-factor	321.920	293	0.118	0.987	0.989	0.016 [.000,0.026]	0.027	Preferred
One-factor	2,056.634	299	< 0.001	0.760	0.739	0.124	–	Poor
Two-factor pre/post	1,468.891	298	< 0.001	0.840	0.826	0.101	–	Poor
Orthogonal four-factor	1,081.847	299	< 0.001	0.893	0.884	0.083	–	Inferior

Additional item-level plausibility checks did not indicate duplicated items or an overhomogeneous item pool. No pairwise item correlation exceeded 90, the maximum exact-response equality rate between item pairs was 475, and no residual correlation exceeded 10. Random split-half checks also retained favorable fit. Taken together, these diagnostics favored the hypothesized measurement structure over single-factor, pre/post, orthogonal, or duplicate-item explanations. Full diagnostic output is reported in Supplementary Appendix B.

### 3.3 Descriptive correlations

The focal variables were positively correlated in theoretically expected directions (Figure 2C). Novelty motivation, face-gain appraisal, event-based social comparison, and sustained positive sport experience showed moderate-to-large positive correlations. Sustained positive sport experience was also positively correlated with subjective vitality, immediate enjoyment, and perceived safety.

### 3.4 Latent structural model

**Figure 3:**
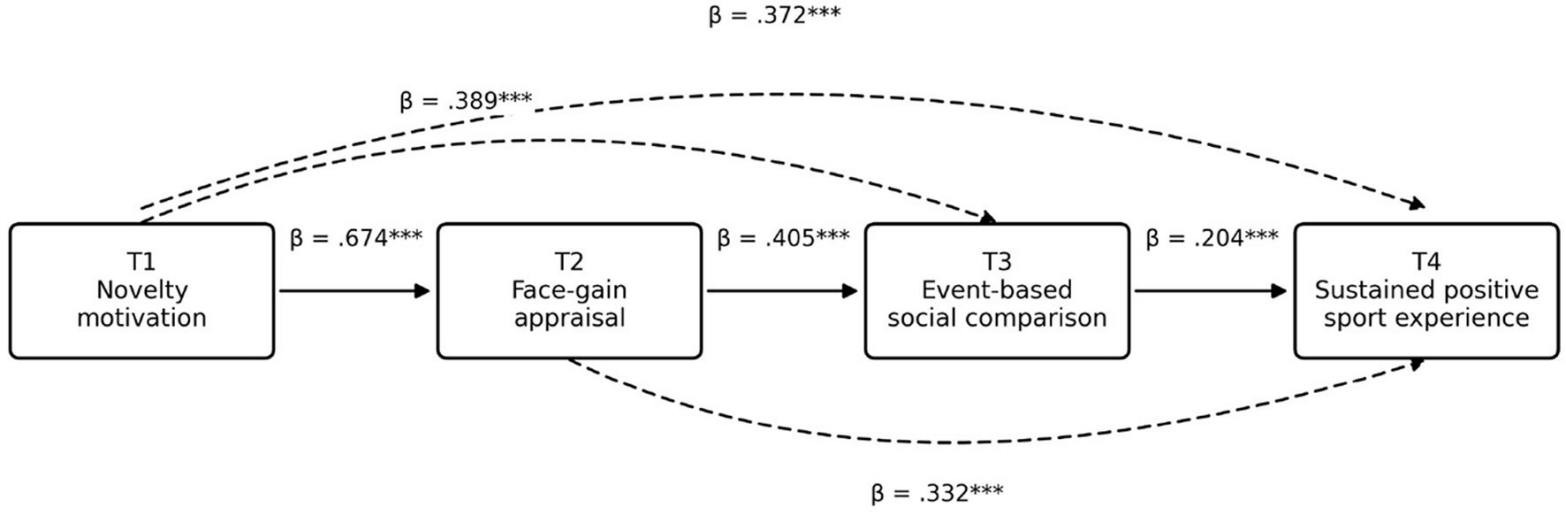
Standardized latent structural estimates for the time-lagged sequential indirect-association model. Values are standardized path coefficients derived from the fitted latent covariance matrix. Solid arrows represent the sequential pathway; dashed arrows represent additional direct paths estimated in the saturated structural model. Indirect estimates are reported in Table 6. n = 385; ***p < 0.001.

The structural portion of the latent model was saturated among the four latent constructs, so the model yielded the same global fit as the four-factor measurement model reported above. These fit indices describe measurement quality rather than comparative evidence that the hypothesized structural ordering outperformed alternative structural specifications. Substantive interpretation focused on the standardized path estimates (Table 4; Figure 3). The sequential paths from novelty motivation to face-gain appraisal, face-gain appraisal to event-based social comparison, and event-based social comparison to sustained positive sport experience were positive. The additional paths from novelty motivation to event-based social comparison and sustained positive sport experience, and from face-gain appraisal to sustained positive sport experience, were also positive.

**Table 4:** Standardized latent structural paths.

Path	β [95% CI]	SE	*z*	*p*	*n*
Novelty motivation to face-gain appraisal	0.674 [0.600,0.748]	0.038	17.848	< 0.001	385
Face-gain appraisal to event-based social comparison	0.405 [0.312,0.498]	0.048	8.515	< 0.001	385
Novelty motivation to event-based social comparison	0.389 [0.296,0.482]	0.048	8.182	< 0.001	385
Event-based social comparison to sustained positive sport experience	0.204 [0.117,0.291]	0.044	4.617	< 0.001	385
Face-gain appraisal to sustained positive sport experience	0.332 [0.244,0.420]	0.045	7.409	< 0.001	385
Novelty motivation to sustained positive sport experience	0.372 [0.285,0.460]	0.045	8.351	< 0.001	385

### 3.5 Indirect associations and sensitivity analyses

**Figure 4:**
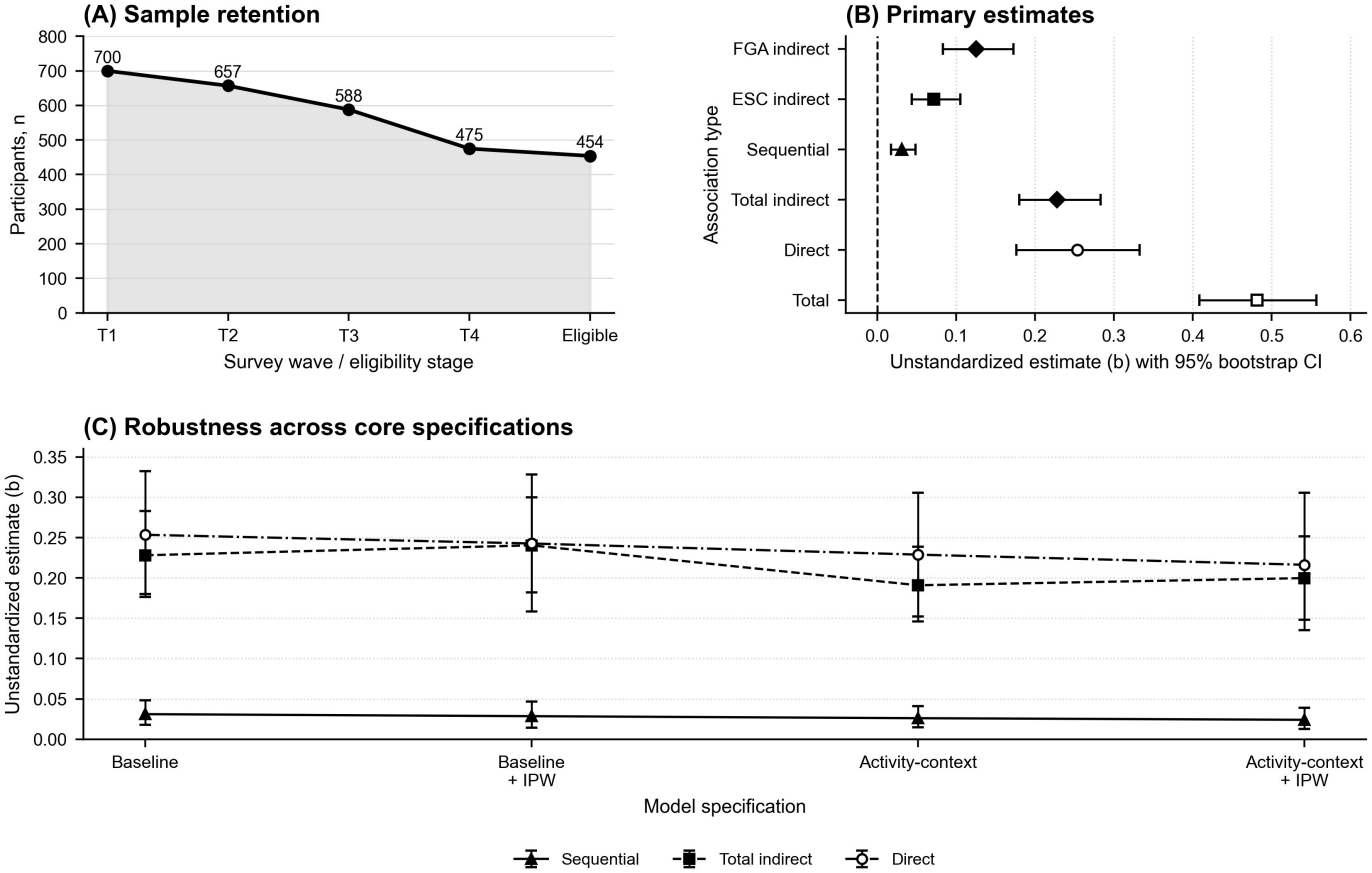
Sample retention, primary indirect-association uncertainty, and robustness. **(A)** Participant retention across the four-wave design and final eligibility screening. Counts represent observed completed records at each wave and were not rounded. **(B)** Primary baseline-covariate adjusted composite-score direct, indirect, and total associations with 5,000 bootstrap 95% confidence intervals (*n* = 454). In panel B, face-gain indirect refers to NM -> FGA -> SPSE, social-comparison indirect refers to NM -> ESC -> SPSE, and sequential indirect refers to NM -> FGA -> ESC -> SPSE. **(C)** Sensitivity profile comparing the sequential indirect, total indirect, and direct point estimates across the primary baseline-covariate model, baseline-covariate IPW model, expanded activity-context sensitivity model, and expanded activity-context IPW model. The supplementary social-media sharing sensitivity model is reported in Table 7 but not plotted in panel C to preserve readability. NM, novelty motivation; FGA, face-gain appraisal; ESC, event-based social comparison; SPSE, sustained positive sport experience; IPW, inverse-probability weighting.

Primary baseline-covariate adjusted composite-score models provided covariate-adjusted estimates for the focal paths (Table 5). Novelty motivation positively predicted face-gain appraisal. In the social-comparison equation, novelty motivation and face-gain appraisal were positive predictors. In the sustained positive sport experience equation, novelty motivation, face-gain appraisal, and event-based social comparison were all positive predictors. Equation R-squared values indicated substantial explained variance across the intermediate and outcome equations. Figure 4A summarizes participant retention across the four-wave design and the final eligibility screening.

**Table 5:** Primary baseline-covariate adjusted composite-score focal path estimates.

Equation	Focal predictor	*b*	HC3 SE	95% CI lower	95% CI upper	*p*	*R^2^*
Face-gain appraisal	Novelty motivation	0.4175	0.0416	0.3359	0.4990	< 0.001	0.425
Social comparison	Novelty motivation	0.3748	0.0501	0.2766	0.4729	< 0.001	0.471
Social comparison	Face-gain appraisal	0.3897	0.0569	0.2782	0.5012	< 0.001	0.471
Sustained positive experience	Novelty motivation	0.2535	0.0404	0.1743	0.3326	< 0.001	0.587
Sustained positive experience	Face-gain appraisal	0.3006	0.0466	0.2092	0.3920	< 0.001	0.587
Sustained positive experience	Event-based social comparison	0.1910	0.0343	0.1237	0.2583	< 0.001	0.587

In the primary baseline-covariate adjusted bootstrap models, confidence intervals excluded zero for all indirect associations (Table 6; Figure 4B). The indirect associations through face-gain appraisal and event-based social comparison were positive, and the sequential indirect association was positive but modest in magnitude. The total indirect association represented approximately 47.4% of the total association, whereas the sequential indirect association represented approximately 6.5%.

**Table 6:** Primary baseline-covariate adjusted composite-score bootstrap indirect associations.

Indirect or total association	Estimate	Boot mean	95% CI lower	95% CI upper	Bootstrap reps
Novelty motivation to face-gain appraisal to sustained positive sport experience	0.1255	0.1255	0.0831	0.1729	5,000
Novelty motivation to event-based social comparison to sustained positive sport experience	0.0716	0.0721	0.0436	0.1054	5,000
Novelty motivation to face-gain appraisal to event-based social comparison to sustained positive sport experience	0.0311	0.0313	0.0177	0.0483	5,000
Total indirect	0.2281	0.2289	0.1801	0.2831	5,000
Direct	0.2535	0.2535	0.1764	0.3326	5,000
Total	0.4816	0.4824	0.4083	0.5568	5,000

Sensitivity estimates were similar in direction and substantive interpretation across the baseline-covariate IPW model, the expanded activity-context sensitivity model, and the expanded activity-context IPW model (Table 7; Figure 4C). Weighting reduced but did not eliminate standardized mean differences between completers and attrited participants on observed T1 predictors. The indirect-association pattern was stable after weighting for observed predictors of completion, and the direct association remained positive across specifications.

**Table 7:** Robustness comparison across covariate, attrition, and social-media specifications.

Specification	Association	Estimate	95% CI lower	95% CI upper	*N*
Baseline covariates	Sequential indirect	0.0311	0.0177	0.0483	454
Baseline covariates	Total indirect	0.2281	0.1801	0.2831	454
Baseline covariates	Direct	0.2535	0.1764	0.3326	454
Baseline + IPW	Sequential indirect	0.0286	0.0145	0.0467	454
Baseline + IPW	Total indirect	0.2404	0.1822	0.3001	454
Baseline + IPW	Direct	0.2427	0.1584	0.3285	454
Activity context	Sequential indirect	0.0261	0.0146	0.0410	454
Activity context	Total indirect	0.1909	0.1462	0.2386	454
Activity context	Direct	0.2289	0.1523	0.3058	454
Activity context + IPW	Sequential indirect	0.0241	0.0125	0.0392	454
Activity context + IPW	Total indirect	0.1998	0.1480	0.2516	454
Activity context + IPW	Direct	0.2163	0.1354	0.3056	454
Social-media sharing	Sequential indirect	0.0305	0.0170	0.0477	454
Social-media sharing	Total indirect	0.2257	0.1772	0.2793	454
Social-media sharing	Direct	0.2522	0.1757	0.3291	454

Because social visibility was part of the theoretical account, a supplementary sensitivity model additionally adjusted the T3 social-comparison and T4 outcome equations for whether participants reported sharing the experience on social media (Table 7). The estimates remained substantively similar, and binary social-media sharing did not reliably predict either social comparison or sustained positive sport experience. This result indicates that the focal pattern was not reducible to whether participants reported sharing the experience on social media.

## 4 Discussion

This study examined how a novel adventure-sport experience remains psychologically positive after the event has ended. In a four-wave sample of urban professional women in China, novelty motivation before highland paragliding was prospectively associated with sustained positive sport experience approximately 1 week later. This association was partly linked with face-gain appraisal immediately after the flight and with event-based social comparison during the post-activity period. The sequential indirect pathway from novelty motivation to face-gain appraisal, social comparison, and sustained positive sport experience also aligned with the data. These findings indicate that the psychological value of a brief adventure-sport activity is not limited to momentary enjoyment during participation; it is also related to how participants interpret the event socially and later compare it with ordinary routines, peer activities, or socially visible alternatives.

### 4.1 Principal findings and sport psychology contribution

The positive association between novelty motivation and sustained positive sport experience is consistent with recent evidence linking motivation, affective experience, and wellbeing in physical activity and sport contexts, including work on autonomous motivation, intrinsic motivation, physical activity, and subjective wellbeing (Courtney et al., 2021; Meyer et al., 2021; White et al., 2024; Meachon et al., 2025; Ntoumanis and Moller, 2025). The finding also extends tourism research on novelty by placing novelty motivation in an embodied sport setting rather than in general travel experience alone (Blomstervik and Olsen, 2022, 2026). Highland paragliding is brief, emotionally salient, physically distinctive, and memorable, which helps contextualize why pre-event novelty motivation remained related to positive experience 1 week after participation.

The direct association between novelty motivation and sustained positive sport experience remained positive after including the two intermediate processes, a pattern consistent with partial indirect association rather than complete statistical explanation. Face-gain appraisal and social comparison represented meaningful components of the prospective association, while other psychological processes remain plausible, including flow, mastery, awe, perceived competence, psychological recovery, and later storytelling. Recent work on tourism and active sport tourism similarly suggests that experiential benefits can continue through recollection, engagement, and recovery after the activity has ended (He et al., 2021; Su et al., 2023; So et al., 2024; Yan et al., 2024; Song et al., 2025). Future studies can test these additional processes in the same time-lagged framework instead of treating post-event experience as a single undifferentiated outcome.

The sport psychology contribution of this study lies in treating sustained positive sport experience as a post-event psychological process rather than as immediate enjoyment or global satisfaction. The four-wave design supports a prospective account in which a brief adventure-sport activity remains positive when pre-event novelty motivation is followed by socially meaningful appraisal and later comparison-based interpretation. This shifts attention from the occurrence of participation to the temporal construction of positive sport meaning.

### 4.2 Social-evaluative and comparative processes

Face-gain appraisal showed the largest single intermediate-process indirect association. This construct captures a culturally embedded form of social-evaluative meaning rather than a narrow concern with vanity or status. In Chinese social contexts, face-related appraisal can reflect visibility, recognition, symbolic accomplishment, and the social interpretation of self-presentation (Shi and Chen, 2024; Wei and Shen, 2025). The construct is not reducible to generic self-presentation terminology, but it functionally overlaps with broader social identity and impression-management processes in which people interpret an activity as reputationally meaningful, identity-signaling, or socially recognizable. Under different terminology, similar processes may appear when sport participation allows individuals to communicate courage, competence, autonomy, lifestyle, or group membership. In the present study, highland paragliding provided a socially legible experience that could signal courage, independence, taste, or a distinctive lifestyle.

Event-based social comparison was also positively associated with sustained positive sport experience and formed part of the prospective association between novelty motivation and the outcome. This finding is consistent with physical activity research showing that comparison effects depend on perceived similarity, identification, and contrast with comparison targets (Perey and Koenigstorfer, 2023; Baga et al., 2024; Arigo et al., 2025). It also aligns with tourism and social media research suggesting that people evaluate their own experiences partly in relation to images, narratives, and social reference points (Su et al., 2021; Xu et al., 2023). In this context, social comparison functioned as an interpretive frame rather than as a uniformly harmful process. After a distinctive sport event, comparison can help participants evaluate the experience as special, meaningful, or personally affirming. The sequential indirect pathway was modest in magnitude but theoretically informative: immediate social-evaluative appraisal was associated with later comparison, and later comparison was associated with retained positive meaning. The supplementary adjustment for binary social-media sharing did not materially change the focal pattern, indicating that sharing status alone did not account for the association. The present measure captured event-based comparison as a broad post-activity process without distinguishing upward, downward, lateral, assimilative, or contrastive comparison; specific comparison targets; platform-specific exposure; interaction volume; feedback quality; or identification vs. contrast. Future research can separate these dimensions and examine whether social media sharing, peer feedback, or perceived similarity with comparison targets strengthens or weakens the association between post-event comparison and sustained positive experience.

### 4.3 Visibility, shareability, and population boundaries

Highland paragliding has contextual features that likely shape how appraisal and comparison unfold. The activity is visually dramatic, aesthetically salient, easily narrated, and often represented through images or video. These features may make the experience more available for social recognition and later comparison than less visible or less shareable adventure sports. The present study did not measure perceived activity visibility, aesthetic salience, platform feedback, audience response, or shareability as moderators. These features should therefore be treated as plausible boundary conditions rather than tested mechanisms.

The mechanisms may operate differently in adventure-sport contexts where the experience is less visually displayable or less socially legible. In such settings, sustained positive experience may depend more strongly on mastery, effort, competence, flow, endurance, risk regulation, or recovery than on face-gain appraisal and social comparison. The findings are also situated in urban professional women in China, a population for whom gendered participation opportunities, social visibility, and culturally situated self-presentation are theoretically relevant. Future studies can test whether the same pattern holds among men, non-binary participants, rural populations, non-professional groups, other age cohorts, and participants in cultural contexts where social recognition, autonomy, and self-presentation carry different meanings.

### 4.4 Practical implications

The findings suggest that psychological value can be supported before, during, and after a brief adventure-sport event. Paragliding providers, sport-tourism agencies, and coaches can strengthen the pre-event phase through clear safety communication, realistic expectation setting, and explicit withdrawal options. These practices allow novelty motivation to remain grounded in informed choice rather than unmanaged anxiety.

After the flight, providers and women's sport organizations can use brief debriefing, credible recognition of accomplishment, and autonomy-supportive reflection prompts to help participants interpret the experience as safely completed, personally chosen, and socially meaningful. Reflective sharing can be encouraged without making visibility or status the primary goal. Recognition-oriented practices should support autonomy, competence, safety, and authentic social connection rather than pressure participants toward risk-taking for external validation.

### 4.5 Limitations and future directions

Several limitations frame interpretation. The study was observational; four-wave timing supports temporal ordering but not experimental causality. The final analytic sample included women who completed the activity and remained in follow-up, excluding registrants who did not fly, withdrew, or had adverse experiences that prevented follow-up. All focal variables were self-reported, and common method bias remains possible (Baumgartner et al., 2021; Baumgartner and Weijters, 2021; Kaltsonoudi et al., 2022; Sturman et al., 2025). Attrition was associated with observed baseline characteristics; IPW sensitivity supported the main pattern, but residual imbalance and unmeasured predictors of completion remain possible. The women-only urban professional sample limits generalization beyond this population.

Measurement and affective-range limits also remain. The adapted measures require independent validation before they can be treated as mature instruments. The social-comparison measure did not separate comparison direction, comparison target, platform exposure, interaction volume, feedback quality, or identification vs. contrast. A simple T2 fear/discomfort indicator was collected, but the study did not systematically assess adverse safety events, dizziness, nausea, physiological arousal, post-flight risk reappraisal, regret, or disappointment. Such negative physiological or affective responses may interrupt the positive appraisal-comparison pathway and should be measured in future time-lagged, experimental, or quasi-experimental designs. Future work can also test flow, awe, mastery, perceived competence, psychological recovery, storytelling, repeat participation, recommendation behavior, and longer-term outdoor recreation engagement.

## 5 Conclusion

In this four-wave observational study, novelty motivation before highland paragliding was prospectively associated with sustained positive sport experience among urban professional women in China. This association was partly linked with face-gain appraisal immediately after the flight and with event-based social comparison during the post-activity period, including a positive sequential indirect association. The study contributes to sport psychology by showing that sustained positive experience after a novel sport event is related not only to the activity itself, but also to the social-evaluative and comparative meanings attached to it over time. These conclusions are framed by the observational self-report design and provide a basis for future longitudinal research on novelty, appraisal, comparison, and positive sport experience.

## Data Availability

The raw data supporting the conclusions of this article will be made available by the authors, without undue reservation.
