# Supplementary material for: Face-gain appraisal and event-based social comparison in the association between novelty motivation and sustained positive sport experience in highland paragliding among urban professional women in China
Source: Front Psychol. 2026 Jun 30;17:1877029. doi: 10.3389/fpsyg.2026.1877029 (PMC13366291; doi:10.3389/fpsyg.2026.1877029)
Supplement: Supplementary file 1 [file Supplementary_file_1.docx]

**Supplementary Appendix A. Measurement Materials and Scoring Rules**

The questionnaire was administered in Chinese; English translations are provided for editorial and peer-review transparency. Unless otherwise noted, items used a 7-point Likert-type response scale from 1 = strongly disagree to 7 = strongly agree; higher composite means indicate higher levels of the construct.

# A1. Scale Adaptation and Content-Validation Procedure

| **Aspect** | **Procedure or rule** |
| --- | --- |
| Construct alignment | The focal measures were written and adapted to represent the study constructs in the highland paragliding context: pre-event novelty motivation, immediate post-event face-gain appraisal, delayed event-based social comparison, and sustained positive sport experience. |
| Contextual adaptation | Item wording was contextualized to recreational highland paragliding while preserving the intended psychological meaning of each construct. The measures should therefore be interpreted as contextualized study measures rather than mature stand-alone clinical or diagnostic instruments. |
| Temporal fit | Items were assigned to waves according to the hypothesized temporal process: T1 before participation, T2 within 1 hour after participation, T3 24-72 hours after participation, and T4 approximately 7 days after participation. |
| Content-validation review | Before analysis, item wording was checked for contextual relevance, temporal fit, clarity, and conceptual alignment with the sport-psychology model reported in the manuscript. No item was reverse-coded in the focal scales. |
| Language and reporting | The questionnaire was administered in Chinese. English translations are provided only to document semantic content for editorial, peer-review, and production assessment. |
| Scoring | Unless otherwise noted, responses used a 7-point Likert-type scale from 1 = strongly disagree to 7 = strongly agree. Composite scores were calculated as item means; higher scores indicate higher construct levels. |

# A2. Focal Measures

| **Timing** | **Code** | **Construct** | **Chineseitemwording** | **Englishtranslation** | **Role** |
| --- | --- | --- | --- | --- | --- |
| T1: 1-3 days before participation | NM1 | Novelty motivation | 我参加高原滑翔伞，是因为它能带来不同于日常生活的体验。 | I participated in highland paragliding because it can provide an experience different from everyday life. | Focal predictor |
| T1: 1-3 days before participation | NM2 | Novelty motivation | 我期待在这项运动中感受到新鲜和未知。 | I expected to feel freshness and uncertainty in this activity. | Focal predictor |
| T1: 1-3 days before participation | NM3 | Novelty motivation | 我希望通过这次体验突破平时熟悉的生活方式。 | I hoped to break away from my familiar everyday lifestyle through this experience. | Focal predictor |
| T1: 1-3 days before participation | NM4 | Novelty motivation | 高原滑翔伞的独特性是吸引我的重要原因。 | The uniqueness of highland paragliding was an important reason it attracted me. | Focal predictor |
| T1: 1-3 days before participation | NM5 | Novelty motivation | 我想体验身体在空中运动带来的刺激感。 | I wanted to experience the excitement of moving through the air. | Focal predictor |
| T1: 1-3 days before participation | NM6 | Novelty motivation | 我希望通过这次活动探索一种新的自我体验。 | I hoped to explore a new self-experience through this activity. | Focal predictor |
| T2: within 1 hour after participation | FGA1 | Face-gain appraisal | 这次体验让我觉得自己的生活方式更具独特性。 | This experience made me feel that my lifestyle was more distinctive. | First mediator |
| T2: within 1 hour after participation | FGA2 | Face-gain appraisal | 这次体验让我感到自己拥有一种值得被认可的经历。 | This experience made me feel that I had an experience worthy of recognition. | First mediator |
| T2: within 1 hour after participation | FGA3 | Face-gain appraisal | 我认为这次活动能够提升他人对我生活品味的看法。 | I believed this activity could improve how others viewed my lifestyle taste. | First mediator |
| T2: within 1 hour after participation | FGA4 | Face-gain appraisal | 完成这次高原滑翔伞后，我感到自己的社会形象有所提升。 | After completing highland paragliding, I felt that my social image had improved. | First mediator |
| T2: within 1 hour after participation | FGA5 | Face-gain appraisal | 这次经历让我觉得自己更符合独立、自信、敢于尝试的形象。 | This experience made me feel closer to an image of being independent, confident, and willing to try new things. | First mediator |
| T2: within 1 hour after participation | FGA6 | Face-gain appraisal | 这次体验让我获得了一种“我做到了”的体面感。 | This experience gave me a sense of dignity from having accomplished it. | First mediator |
| T3: 24-72 hours after participation | ESC1 | Event-based social comparison | 活动结束后，我会将这次经历与他人的运动或旅行经历进行比较。 | After the activity, I compared this experience with other people's sport or travel experiences. | Second mediator |
| T3: 24-72 hours after participation | ESC2 | Event-based social comparison | 我会想到自己是否拥有比身边人更独特的体验。 | I thought about whether I had a more distinctive experience than people around me. | Second mediator |
| T3: 24-72 hours after participation | ESC3 | Event-based social comparison | 当看到他人的休闲或运动经历时，我会联想到这次滑翔伞体验。 | When seeing other people's leisure or sport experiences, I thought of this paragliding experience. | Second mediator |
| T3: 24-72 hours after participation | ESC4 | Event-based social comparison | 这次经历让我在与他人的比较中感到某种优势。 | This experience made me feel some advantage in comparison with others. | Second mediator |
| T3: 24-72 hours after participation | ESC5 | Event-based social comparison | 这次经历也激励我以后尝试更有挑战性的活动。 | This experience also motivated me to try more challenging activities in the future. | Second mediator |
| T3: 24-72 hours after participation | ESC6 | Event-based social comparison | 我会关注他人如何评价或回应我的这次体验。 | I paid attention to how others evaluated or responded to this experience. | Second mediator |
| T4: approximately 7 days after participation | SPSE1 | Sustained positive sport experience | 回想这次高原滑翔伞经历时，我仍然感到愉悦。 | When recalling this highland paragliding experience, I still felt pleased. | Outcome |
| T4: approximately 7 days after participation | SPSE2 | Sustained positive sport experience | 这次经历在活动结束后仍给我留下积极感受。 | This experience continued to leave me with positive feelings after the activity ended. | Outcome |
| T4: approximately 7 days after participation | SPSE3 | Sustained positive sport experience | 这次经历让我觉得自己的生活更有活力。 | This experience made me feel that my life had more vitality. | Outcome |
| T4: approximately 7 days after participation | SPSE4 | Sustained positive sport experience | 这次经历让我感到精神上更加充实。 | This experience made me feel more fulfilled psychologically. | Outcome |
| T4: approximately 7 days after participation | SPSE5 | Sustained positive sport experience | 这次经历让我对自己的勇气或能力有更积极的看法。 | This experience gave me a more positive view of my courage or ability. | Outcome |
| T4: approximately 7 days after participation | SPSE6 | Sustained positive sport experience | 这次经历对我而言不仅是一次消费，也是一段有意义的运动体验。 | For me, this experience was not only a consumer activity but also a meaningful sport experience. | Outcome |
| T4: approximately 7 days after participation | SPSE7 | Sustained positive sport experience | 一周后回想起来，我仍认为这次体验值得。 | Looking back one week later, I still considered this experience worthwhile. | Outcome |
| T4: approximately 7 days after participation | SPSE8 | Sustained positive sport experience | 这次经历增强了我继续参与户外或冒险运动的兴趣。 | This experience strengthened my interest in continuing to participate in outdoor or adventure sports. | Outcome |

# A3. Baseline Psychological Covariates

| **Timing** | **Code** | **Construct** | **Chineseitemwording** | **Englishtranslation** | **Role** |
| --- | --- | --- | --- | --- | --- |
| T1 | SS1 | Sensation Seeking | 我通常愿意尝试新鲜、有刺激感的活动。 | I am generally willing to try new and exciting activities. | Baseline covariate |
| T1 | SS2 | Sensation Seeking | 我喜欢能让我感到兴奋和挑战的体验。 | I like experiences that make me feel excited and challenged. | Baseline covariate |
| T1 | SS3 | Sensation Seeking | 如果条件安全可控，我愿意尝试有一定风险的运动。 | If conditions are safe and controllable, I am willing to try sports with some risk. | Baseline covariate |
| T1 | RP1 | Risk Perception | 我认为高原滑翔伞具有一定风险。 | I think highland paragliding involves some risk. | Baseline covariate |
| T1 | RP2 | Risk Perception | 我对这次活动的安全性有所担心。 | I had some concerns about the safety of this activity. | Baseline covariate |
| T1 | RP3 | Risk Perception | 我会在参与前认真评估这项活动的风险。 | Before participating, I carefully evaluated the risks of this activity. | Baseline covariate |
| T1 | BPA1 | Baseline Positive Affect | 最近几天，我总体感到积极。 | In the past few days, I generally felt positive. | Baseline covariate |
| T1 | BPA2 | Baseline Positive Affect | 最近几天，我感到精力较好。 | In the past few days, I felt relatively energetic. | Baseline covariate |
| T1 | BPA3 | Baseline Positive Affect | 最近几天，我对生活中的事情较有兴趣。 | In the past few days, I was interested in things in life. | Baseline covariate |
| T1 | BFC1 | Baseline Face Concern | 我在意重要他人如何看待我的生活方式。 | I care about how important others view my lifestyle. | Baseline covariate |
| T1 | BFC2 | Baseline Face Concern | 我希望自己的经历能体现良好的社会形象。 | I hope my experiences can reflect a favorable social image. | Baseline covariate |
| T1 | BFC3 | Baseline Face Concern | 我会关注自己的行为是否能获得他人的尊重。 | I pay attention to whether my behavior can gain others' respect. | Baseline covariate |
| T1 | BSCO1 | Baseline Social Comparison Orientation | 我有时会把自己的经历与他人的经历进行比较。 | I sometimes compare my experiences with other people's experiences. | Baseline covariate |
| T1 | BSCO2 | Baseline Social Comparison Orientation | 我会关注他人在类似情境中的选择和表现。 | I pay attention to other people's choices and performance in similar situations. | Baseline covariate |
| T1 | BSCO3 | Baseline Social Comparison Orientation | 我会通过比较来判断自己的体验是否特别。 | I use comparison to judge whether my own experience is distinctive. | Baseline covariate |

# A4. Validation, Activity-Context, and Additional Measures

| **Timing** | **Code** | **Construct** | **Chineseitemwording** | **Englishtranslation** | **Role** |
| --- | --- | --- | --- | --- | --- |
| T2 | IE1 | Immediate Enjoyment | 刚完成这次活动时，我感到愉快。 | Immediately after completing the activity, I felt happy. | Validation/activity-context covariate |
| T2 | IE2 | Immediate Enjoyment | 这次活动让我感到开心。 | This activity made me feel pleased. | Validation/activity-context covariate |
| T2 | IE3 | Immediate Enjoyment | 我享受刚刚完成的滑翔伞体验。 | I enjoyed the paragliding experience I had just completed. | Validation/activity-context covariate |
| T2 | ACH1 | Achievement Experience | 完成这次活动让我有成就感。 | Completing this activity gave me a sense of achievement. | Additional post-activity measure |
| T2 | ACH2 | Achievement Experience | 我为自己完成这次体验感到自豪。 | I felt proud of myself for completing this experience. | Additional post-activity measure |
| T2 | ACH3 | Achievement Experience | 这次经历让我觉得自己克服了一项挑战。 | This experience made me feel that I had overcome a challenge. | Additional post-activity measure |
| T2 | FLOW1 | Flow State | 在活动过程中，我能专注于当下体验。 | During the activity, I could focus on the present experience. | Additional post-activity measure |
| T2 | FLOW2 | Flow State | 在活动过程中，我暂时忘记了日常烦恼。 | During the activity, I temporarily forgot everyday worries. | Additional post-activity measure |
| T2 | FLOW3 | Flow State | 在活动过程中，我感到自己沉浸其中。 | During the activity, I felt immersed in it. | Additional post-activity measure |
| T2 | PS1 | Perceived Safety | 我在活动过程中总体感到安全。 | Overall, I felt safe during the activity. | Activity-context covariate/validation variable |
| T2 | PS2 | Perceived Safety | 教练或工作人员让我感到放心。 | The coach or staff made me feel reassured. | Activity-context covariate/validation variable |
| T2 | PS3 | Perceived Safety | 活动组织和设备让我感到可靠。 | The organization and equipment of the activity felt reliable. | Activity-context covariate/validation variable |
| T3 | PSR1 | Perceived Social Recognition | 我感觉他人对这次经历表现出兴趣。 | I felt that others showed interest in this experience. | Additional social-response measure |
| T3 | PSR2 | Perceived Social Recognition | 我感觉这次经历容易获得他人的积极评价。 | I felt that this experience was likely to receive positive evaluation from others. | Additional social-response measure |
| T3 | PSR3 | Perceived Social Recognition | 我认为这次经历增强了我在社交圈中的话题性。 | I believed this experience increased my topicality in my social circle. | Additional social-response measure |
| T3 | PSR4 | Perceived Social Recognition | 我感到这次经历让我被看见或被认可。 | I felt that this experience made me seen or recognized. | Additional social-response measure |
| T4 | SV1 | Subjective Vitality | 最近几天，我感到自己更有精力。 | In the past few days, I felt more energetic. | Validation variable |
| T4 | SV2 | Subjective Vitality | 最近几天，我感到生活状态更有活力。 | In the past few days, I felt that my life state had more vitality. | Validation variable |
| T4 | SV3 | Subjective Vitality | 最近几天，我感到精神上比较充沛。 | In the past few days, I felt mentally vigorous. | Validation variable |
| T4 | MF1 | Meaningfulness | 这次经历让我觉得生活中可以有更多新的可能。 | This experience made me feel that life can have more new possibilities. | Additional outcome-related measure |
| T4 | MF2 | Meaningfulness | 这次经历让我对自己有了更积极的理解。 | This experience gave me a more positive understanding of myself. | Additional outcome-related measure |
| T4 | MF3 | Meaningfulness | 这次经历对我来说具有一定的个人意义。 | This experience had a certain personal meaning for me. | Additional outcome-related measure |
| T4 | RI1 | Reparticipation Intention | 如果条件允许，我愿意再次参加类似活动。 | If conditions allow, I am willing to participate in similar activities again. | Additional outcome-related measure |
| T4 | RI2 | Reparticipation Intention | 我愿意向他人推荐高原滑翔伞体验。 | I am willing to recommend the highland paragliding experience to others. | Additional outcome-related measure |
| T4 | RI3 | Reparticipation Intention | 我未来愿意继续尝试户外或冒险运动。 | In the future, I am willing to continue trying outdoor or adventure sports. | Additional outcome-related measure |

# A5. Demographic, Experience, Context, and Social-Media Variables

| **Timing** | **Variable** | **Meaning** | **Use in manuscript** |
| --- | --- | --- | --- |
| T1 | age | Age in years | Continuous variable. Used as a baseline covariate. |
| T1 | education | Highest education level | Categorical variable. Used as a baseline covariate. |
| T1 | income | Monthly income category | Categorical variable. Used as a baseline covariate. |
| T1 | occupation_type | Occupation type | Categorical background variable. |
| T1 | career_level | Career level | Categorical variable. Used as a baseline covariate. |
| T1 | married | Marital status | Binary variable reported in Table 1. |
| T1 | has_child | Parenthood status | Binary variable reported in Table 1. |
| T1 | city_type | City category | First-tier/new first-tier, provincial capital, prefecture-level, or other city; reported in Table 1. |
| T1 | first_paragliding | First highland paragliding experience | Binary variable reported in Table 1 and considered in sensitivity/covariate work. |
| T1 | prior_adventure_sport | Prior adventure-sport experience | Baseline covariate. |
| T1 | sport_frequency | Sport-participation frequency | Baseline covariate. |
| T2 | flight_site | Paragliding site | Five site categories S01-S05; reported in Table 1. |
| T2 | flight_duration | Flight duration in minutes | Continuous activity-context covariate; mean and SD reported in Table 1. |
| T2 | weather_satisfaction | Weather satisfaction | Activity-context sensitivity covariate. |
| T2 | coach_service | Coach/staff service evaluation | Activity-context sensitivity covariate. |
| T2 | completed_flight | Completed flight as planned | Screening/context variable. |
| T2 | fear_discomfort | Fear or physical discomfort | Context variable. |
| T2 | photo_video | Photo/video recording | Context variable. |
| T3 | shared_social_media | Social-media sharing after the event | Binary variable used for the social-media sharing sensitivity analysis and reported in Table 1. |
| T3 | platform | Sharing platform | WeChat Moments, Xiaohongshu, Douyin, Weibo, or other platform. |
| T3 | interactions | Approximate social-media interactions | Approximate likes, comments, or interactions after sharing. |
| T3 | feedback_valence | Overall feedback valence | Positive, neutral, or negative feedback. |
| T4 | followup_interval | Follow-up interval in days | Continuous variable; mean and SD reported in Table 1. |

# A6. Scoring, Missingness, and Attention Checks

| **Element** | **Rule or wording** |
| --- | --- |
| Composite scoring | Multi-item variables were scored as item means when valid item responses were available; higher scores indicate higher construct levels. No reverse-coded focal items were used. |
| Reliability reporting | Internal consistency was reported by Cronbach alpha, McDonald omega, composite reliability, and AVE for the four focal constructs. |
| Scale-specific sample sizes | Reliability and validity sample sizes vary by scale because construct-level estimates required available responses for the relevant item set. |
| Attention check | The attention-check instruction was: 'To confirm that you are responding carefully, please select somewhat agree.' Participants who failed the required checks across waves were excluded from the final eligible analytic sample. |
| Adaptation note | These instruments were contextualized for this study and are not presented as mature clinical or diagnostic measures. Interpretation relies on the reliability, CFA, convergent-validity, discriminant-validity, and sensitivity evidence reported in the manuscript and Supplementary Appendix B. |
