# Supplementary material for: Face-gain appraisal and event-based social comparison in the association between novelty motivation and sustained positive sport experience in highland paragliding among urban professional women in China
Source: Front Psychol. 2026 Jun 30;17:1877029. doi: 10.3389/fpsyg.2026.1877029 (PMC13366291; doi:10.3389/fpsyg.2026.1877029)
Supplement: Supplementary file 2 [file Supplementary_file_2.docx]

**Supplementary Appendix B. Statistical Reproducibility, Diagnostics, and Sensitivity Materials**

# B1. Manuscript-Appendix Matching Summary

| **Component** | **Current production value** | **Match status** |
| --- | --- | --- |
| Manuscript sample flow | T1 = 700; T2 = 657; T3 = 588; T4 = 475; excluded attention-check failures = 21; final eligible N = 454. | Matches latest manuscript Methods, Figure 4A, and Results. |
| Primary CFA/latent SEM sample | CFA, HTMT, and latent structural models used complete information on the focal item set with n = 385. | Matches Table 3, Figure 3, and latent SEM reporting. |
| Adjusted composite mediation sample | Regression-based mediation and robustness models used the final eligible analytic sample, N = 454. | Matches Tables 5-7. |
| Measurement model | Correlated four-factor CFA: chi-square(293) = 321.920, p = .118, CFI = .987, TLI = .989, RMSEA = .016 [.000, .026], SRMR = .027. | Matches Table 3. |
| Appendix A source | Item wording comes from the local four-wave questionnaire file and uses the manuscript's stated Chinese administration language and scoring approach. | Matches Methods measurement description. |

# B2. Four-Wave Sample Flow and Timing Checks

| **Stage** | **Participants** | **Definition** |
| --- | --- | --- |
| T1 respondents | 700 | Baseline survey 1-3 days before participation. |
| T2 completed | 657 | Within 1 hour after participation. |
| T3 completed | 588 | 24-72 hours after participation. |
| T4 completed | 475 | Approximately 7 days after participation. |
| Excluded after attention checks | 21 | Participants not passing all four required attention checks. |
| Final eligible analytic sample | 454 | Primary composite-based adjusted analyses and descriptive Table 1. |
| **Timing/material check** | **Value** | **Use** |
| Field period | 2025-10-01 to 2025-12-10 |  |
| T4 follow-up interval | M = 8.00 days, SD = 0.83 | Reported in Table 1. |
| Mean flight duration | M = 16.91 minutes, SD = 6.63 | Reported in Table 1 and used as an activity-context sensitivity covariate. |

# B3. Descriptive Characteristics Reported in Table 1

| **Characteristic** | **N (%) or M (SD)** |
| --- | --- |
| Age, years | 31.36 (6.13) |
| Flight duration, minutes | 16.91 (6.63) |
| Follow-up interval, days | 8.00 (0.83) |
| First paragliding experience: yes | 317 (69.8%) |
| First paragliding experience: no | 137 (30.2%) |
| Shared the experience on social media: yes | 239 (52.6%) |
| Shared the experience on social media: no | 215 (47.4%) |
| Married: yes | 198 (43.6%) |
| Married: no | 256 (56.4%) |
| Has child: yes | 102 (22.5%) |
| Has child: no | 352 (77.5%) |
| First-tier/new first-tier city | 168 (37.0%) |
| Provincial capital city | 137 (30.2%) |
| Prefecture-level city | 109 (24.0%) |
| Other city | 40 (8.8%) |
| Paragliding site S01 | 106 (23.3%) |
| Paragliding site S02 | 83 (18.3%) |
| Paragliding site S03 | 127 (28.0%) |
| Paragliding site S04 | 78 (17.2%) |
| Paragliding site S05 | 60 (13.2%) |

# B4. Reliability and Convergent Validity

| **Construct** | **Items** | **Complete item n** | **Alpha** | **Omega** | **CR** | **AVE** |
| --- | --- | --- | --- | --- | --- | --- |
| Novelty motivation | 6 | 445 | .922 | .922 | .926 | .677 |
| Face-gain appraisal | 6 | 433 | .894 | .895 | .895 | .589 |
| Event-based social comparison | 6 | 432 | .911 | .912 | .913 | .636 |
| Sustained positive sport experience | 8 | 426 | .938 | .938 | .940 | .664 |

Interpretation: Internal consistency was high for all four focal constructs. AVE for face-gain appraisal was below .60 but remained above the conventional .50 minimum; this supports convergent validity while avoiding overstatement.

# B5. CFA Model Comparison and Plausibility Diagnostics

| **Model** | **χ²** | **df** | **p** | **CFI** | **TLI** | **RMSEA[90% CI]** | **SRMR** | **Fit** |
| --- | --- | --- | --- | --- | --- | --- | --- | --- |
| Correlated four-factor | 321.920 | 293 | .118 | .987 | .989 | .016 [.000, .026] | .027 | Preferred |
| One-factor | 2056.634 | 299 | < .001 | .760 | .739 | .124 | -- | Poor |
| Two-factor pre/post | 1468.891 | 298 | < .001 | .840 | .826 | .101 | -- | Poor |
| Orthogonal four-factor | 1081.847 | 299 | < .001 | .893 | .884 | .083 | -- | Inferior |
| **Diagnostic** | | | **Manuscript value/check** | | | **Implication** | | |
| Pairwise item correlations | | | No pairwise item correlation exceeded .90. | | | Reduces concern that unusually favorable fit was produced by duplicate or near-duplicate items. | | |
| Exact-response equality | | | Maximum exact-response equality was .475. | | | Does not suggest item duplication or mechanical response copying. | | |
| Residual correlations | | | No residual correlation exceeded .10 in the preferred four-factor CFA. | | | Supports the claim that local dependence did not drive fit. | | |
| Split-half check | | | Random split-half CFA checks retained favorable fit. | | | Supports robustness of the measurement solution, while not proving population invariance. | | |
| Model comparison | | | One-factor, two-factor pre/post, and orthogonal four-factor models fit substantially worse than the correlated four-factor model. | | | Supports discriminant structure among the four focal constructs. | | |

# B6. Latent Structural Estimates

| **Path** | **β [95% CI]** | **SE** | **z** | **p** | **n** |
| --- | --- | --- | --- | --- | --- |
| Novelty motivation to face-gain appraisal | .674 [.600, .748] | .038 | 17.848 | < .001 | 385 |
| Face-gain appraisal to event-based social comparison | .405 [.312, .498] | .048 | 8.515 | < .001 | 385 |
| Novelty motivation to event-based social comparison | .389 [.296, .482] | .048 | 8.182 | < .001 | 385 |
| Event-based social comparison to sustained positive sport experience | .204 [.117, .291] | .044 | 4.617 | < .001 | 385 |
| Face-gain appraisal to sustained positive sport experience | .332 [.244, .420] | .045 | 7.409 | < .001 | 385 |
| Novelty motivation to sustained positive sport experience | .372 [.285, .460] | .045 | 8.351 | < .001 | 385 |

# B7. Covariate-Adjusted Composite Regression Models

| **Equation** | **Focal predictor** | **b** | **HC3 SE** | **95% CI lower** | **95% CI upper** | **p** | **R²** |
| --- | --- | --- | --- | --- | --- | --- | --- |
| Face-gain appraisal | Novelty motivation | 0.4175 | 0.0416 | 0.3359 | 0.4990 | < .001 | .425 |
| Social comparison | Novelty motivation | 0.3748 | 0.0501 | 0.2766 | 0.4729 | < .001 | .471 |
| Social comparison | Face-gain appraisal | 0.3897 | 0.0569 | 0.2782 | 0.5012 | < .001 | .471 |
| Sustained positive experience | Novelty motivation | 0.2535 | 0.0404 | 0.1743 | 0.3326 | < .001 | .587 |
| Sustained positive experience | Face-gain appraisal | 0.3006 | 0.0466 | 0.2092 | 0.3920 | < .001 | .587 |
| Sustained positive experience | Event-based social comparison | 0.1910 | 0.0343 | 0.1237 | 0.2583 | < .001 | .587 |

Model note: The composite-score models adjusted for baseline demographic, experience, and psychological covariates described in the manuscript and Appendix A. HC3 robust standard errors were reported for regression coefficients.

# B8. Bootstrap Indirect Effects

| **Indirect or total association** | **Estimate** | **Boot mean** | **95% CI lower** | **95% CI upper** | **Bootstrap reps** |
| --- | --- | --- | --- | --- | --- |
| Novelty motivation to face-gain appraisal to sustained positive sport experience | 0.1255 | 0.1255 | 0.0831 | 0.1729 | 5000 |
| Novelty motivation to event-based social comparison to sustained positive sport experience | 0.0716 | 0.0721 | 0.0436 | 0.1054 | 5000 |
| Novelty motivation to face-gain appraisal to event-based social comparison to sustained positive sport experience | 0.0311 | 0.0313 | 0.0177 | 0.0483 | 5000 |
| Total indirect | 0.2281 | 0.2289 | 0.1801 | 0.2831 | 5000 |
| Direct | 0.2535 | 0.2535 | 0.1764 | 0.3326 | 5000 |
| Total | 0.4816 | 0.4824 | 0.4083 | 0.5568 | 5000 |

Bootstrap note: Indirect-effect confidence intervals were based on 5,000 nonparametric bootstrap resamples. Effects are reported on the composite-score metric used in the regression-based mediation models.

# B9. Robustness and Sensitivity Analyses

| **Specification** | **Association** | **Estimate** | **95% CI lower** | **95% CI upper** | **N** |
| --- | --- | --- | --- | --- | --- |
| Baseline covariates | Sequential indirect | 0.0311 | 0.0177 | 0.0483 | 454 |
| Baseline covariates | Total indirect | 0.2281 | 0.1801 | 0.2831 | 454 |
| Baseline covariates | Direct | 0.2535 | 0.1764 | 0.3326 | 454 |
| Baseline + IPW | Sequential indirect | 0.0286 | 0.0145 | 0.0467 | 454 |
| Baseline + IPW | Total indirect | 0.2404 | 0.1822 | 0.3001 | 454 |
| Baseline + IPW | Direct | 0.2427 | 0.1584 | 0.3285 | 454 |
| Activity context | Sequential indirect | 0.0261 | 0.0146 | 0.0410 | 454 |
| Activity context | Total indirect | 0.1909 | 0.1462 | 0.2386 | 454 |
| Activity context | Direct | 0.2289 | 0.1523 | 0.3058 | 454 |
| Activity context + IPW | Sequential indirect | 0.0241 | 0.0125 | 0.0392 | 454 |
| Activity context + IPW | Total indirect | 0.1998 | 0.1480 | 0.2516 | 454 |
| Activity context + IPW | Direct | 0.2163 | 0.1354 | 0.3056 | 454 |
| Social-media sharing | Sequential indirect | 0.0305 | 0.0170 | 0.0477 | 454 |
| Social-media sharing | Total indirect | 0.2257 | 0.1772 | 0.2793 | 454 |
| Social-media sharing | Direct | 0.2522 | 0.1757 | 0.3291 | 454 |

# B10. Correlations Among Focal and Validation Variables

|  | **NM** | **FGA** | **ESC** | **SPSE** | **SV** | **IE** | **PS** |
| --- | --- | --- | --- | --- | --- | --- | --- |
| NM | 1.00 | .59 | .58 | .66 | .34 | .37 | .07 |
| FGA | .59 | 1.00 | .59 | .65 | .33 | .37 | .09 |
| ESC | .58 | .59 | 1.00 | .60 | .26 | .24 | .05 |
| SPSE | .66 | .65 | .60 | 1.00 | .50 | .43 | .15 |
| SV | .34 | .33 | .26 | .50 | 1.00 | .33 | .14 |
| IE | .37 | .37 | .24 | .43 | .33 | 1.00 | .35 |
| PS | .07 | .09 | .05 | .15 | .14 | .35 | 1.00 |

Abbreviations: NM = novelty motivation; FGA = face-gain appraisal; ESC = event-based social comparison; SPSE = sustained positive sport experience; SV = subjective vitality; IE = immediate enjoyment; PS = perceived safety. Values correspond to the current Figure 2 correlation panel and are rounded to two decimals.

# B11. Software Versions

| **Package** | **Version** |
| --- | --- |
| Python | 3.13.3 |
| pandas | 2.3.3 |
| NumPy | 2.3.5 |
| SciPy | 1.17.0 |
| statsmodels | 0.14.6 |
| semopy | 2.3.11 |
